# Genomic Diversity and Runs of Homozygosity in Bernese Mountain Dogs

**DOI:** 10.3390/genes14030650

**Published:** 2023-03-04

**Authors:** Anna Letko, Benoît Hédan, Anna Snell, Alexander C. Harris, Vidhya Jagannathan, Göran Andersson, Bodil S. Holst, Elaine A. Ostrander, Pascale Quignon, Catherine André, Tosso Leeb

**Affiliations:** 1 Institut de Génétique et Développement de Rennes (IGDR)–UMR6290, University Rennes 1, CNRS-INSERM, 35000 Rennes, France; 2 Department of Clinical Sciences, Swedish University of Agricultural Sciences (SLU), 750 07 Uppsala, Sweden; 3 National Human Genome Research Institute, National Institutes of Health, Bethesda, MD 20984, USA; 4 Institute of Genetics, Vetsuisse Faculty, University of Bern, 3012 Bern, Switzerland; 5 Department of Animal Breeding and Genetics, Swedish University of Agricultural Sciences (SLU), 750 07 Uppsala, Sweden

**Keywords:** population structure, inbreeding, whole-genome sequencing, immune system, cancer

## Abstract

Bernese mountain dogs are a large dog breed formed in the early 1900s in Switzerland. While originally farm dogs that were used for pulling carts, guarding, and driving cattle, today they are considered multi-purpose companion and family dogs. The breed is predisposed to several complex diseases, such as histiocytic sarcoma, degenerative myelopathy, or hip dysplasia. Using whole-genome sequencing (WGS) data, we assessed the genomic architecture of 33 unrelated dogs from four countries: France, Sweden, Switzerland, and the United States. Analysis of runs of homozygosity (ROH) identified 12,643 ROH with an average length of 2.29 Mb and an average inbreeding coefficient of 0.395. Multidimensional scaling analysis of the genetic relatedness revealed limited clustering of European versus USA dogs, suggesting exchanges of breeding stock between continents. Furthermore, only two mtDNA haplotypes were detected in the 33 studied dogs, both of which are widespread throughout multiple dog breeds. WGS-based ROH analyses revealed several fixed or nearly fixed regions harboring discreet morphological trait-associated as well as disease-associated genetic variants. Several genes involved in the regulation of immune cells were found in the ROH shared by all dogs, which is notable in the context of the breed’s strong predisposition to hematopoietic cancers. High levels of inbreeding and relatedness, strongly exaggerated in the last 30 years, have likely led to the high prevalence of specific genetic disorders in this breed.

## 1. Introduction

Bernese mountain dogs are a popular dog breed developed in the last hundred years using strong selection of long-haired tricolored all-purpose working farm dogs, as documented in the breed’s standard by the Fédération Cynologique Internationale [1]. According to available information from the respective national breeding clubs, the current population sizes in Europe are approximately 23,000 in France, 2700 in Sweden, and 8000 in Switzerland, and the popularity of the breed is stable or slightly decreasing based on the average number of registered dogs per year over the last five years (2018–2022 registrations in France: 3271, 3210, 3183, 3352, 2935; Sweden: 383, 393, 398, 347, 318; Switzerland: 305, 364, 282, 306, 247). In the United States, the popularity of the breed reportedly increased from 25th place in 2017 to 20th place in 2021 as ranked by the American Kennel Club (https://www.akc.org/most-popular-breeds/, accessed on 22 February 2023). Originally, these dogs were used for guarding farms, pulling carts, and driving cattle in the mountain regions of Switzerland. Due to their versatility, they are now globally known and appreciated as companion and family dogs but are also used for various dog sports and activities such as obedience, tracking, cart pulling, water rescue, or canis therapy. [2].

The breed is known to have a high prevalence of several complex diseases, such as histiocytic sarcoma (OMIA 000620-9615), degenerative myelopathy (OMIA 000263-9615), and hip dysplasia (OMIA 000473-9615), all of which pose significant concern to the dog owners and contribute to decreased quality of life and shorter lifespan for affected dogs [3,4,5,6,7]. A high incidence of hematopoietic cancers was also reported in Flat-coated retriever and Rottweiler [8]. High cancer-related mortality has been reported in Flat-coated retriever (54.3%), Bernese mountain dog (45.7%), and Rottweiler (45.3%), while the lowest frequency of death due to cancer was found in Shih-tzu (14.5%), German spitz (16.3%), and Dachshund (16.7%) [9,10]. Abadie et al. [11] performed an extensive genealogical study of more than 20,000 Bernese mountain dogs from France that showed a popular sire effect in the breed. Only 5.5% of males in the large multigenerational pedigree were used for reproduction at each generation, and less than 1% of sires produced about half of the next generation offspring in that group of French dogs [11].

The population history of emerging dog breeds together with the current management strategies in dog breeding is increasingly recognized as the cause of reduced genetic diversity, impaired health, and animal welfare issues [12]. In recent years, many dog breeds have been studied in regard to within-breed diversity using single nucleotide polymorphism (SNP)-based genotypes using arrays (e.g., German shorthaired pointers [13], Braque Français [14], Bullmastiff dogs [15], or Border collies [16]), and/or whole-genome sequencing (WGS) data (e.g., Standard poodles [17], Norwegian Lundehund dogs [18], or Leonbergers [19]). Most studies utilize analyses of runs of homozygosity to quantify the extent of genomic inbreeding, to explore breed-specific regions of variation, or to gain insights into the breed’s population history.

In this study, we aimed to analyze WGS data of 33 unrelated Bernese mountain dogs originating from France, Sweden, Switzerland, and the United States in order to assess the genetic diversity within the breed.

## 2. Materials and Methods

### 2.1. Samples

EDTA blood samples from 33 Bernese mountain dogs were collected for genomic DNA isolation from France (*n* = 7), Sweden (*n* =7), Switzerland (*n* = 7), and the United States (*n* = 12). All dogs were unrelated for at least three generations according to the available pedigree information, and all were reportedly healthy at time of sampling. All samples were collected according to local regulations by veterinarians with the owner’s consent in the course of the dogs’ medical care and submitted to local biobanks: Cani-DNA BRC at the University of Rennes in France [20], Swedish University of Agricultural Sciences in Sweden, the Vetsuisse Biobank at the University of Bern in Switzerland, and National Human Genome Research Institute in the United States.

### 2.2. Whole-Genome Sequence Data

Illumina paired-end 2 × 150 bp whole-genome sequencing of the 33 Bernese mountain dogs was performed after preparation of PCR-free libraries. Publicly available sequencing data previously generated through the international projects of the Dog10K Consortium [21] and the Dog Biomedical Variant Database Consortium [22] were also used for this study. Fastq-files were quality filtered with fastp v 0.19.5 [23] and aligned to the dog reference genome assembly UU_Cfam_GSD_1.0 (GCF_011100685.1) [24] using the bwa-mem2 v 2.2.0 algorithm [25]. Variant calling of single nucleotide variants (SNV) and small indels was performed following the best practices pipeline established for the Genome Analysis Toolkit v4 [26]. A total of 12,814,168 variants were called. The average coverage of the 33 genomes was 23.7× (ranging from 9.4× to 50.6×). The sequence data were deposited at the European Nucleotide Archive; accession numbers and further details are available in Supplementary Table S1. Additionally, variability of the mitochondrial genome was explored to determine the diversity in mtDNA haplotypes of the studied dogs using the nomenclature described by both Pereira et al. [27] and Duleba et al. [28]. The Integrative Genomics Viewer was used to visually inspect and confirm the detected variants [29].

### 2.3. Population Structure

Multidimensional scaling (MDS) was generated with PLINK v1.9 [30]. The initial dataset of 12,814,168 variants was filtered to determine high-quality (using the PASS filter and call rate >90%) biallelic SNVs on the 38 canine autosomes, leaving a set of 6,344,366 markers. These were further pruned for linkage disequilibrium (LD). The LD parameters included a window size of 1000 markers with step size of 100 and pairwise r^2^ threshold of 0.8. In total, 274,240 markers were left, and the dimension reduction based on raw Hamming distances as implemented in PLINK v1.9 was performed. The first three MDS components were extracted for visualizing genetic distances in the R environment v4.2.1 [31].

### 2.4. Runs of Homozygosity

PLINK v1.9 [30] was used for quality control pruning of the initial 12,814,168 variants called in all 33 dogs as well as the runs of homozygosity (ROH) analyses. Only high-quality (using the PASS filter and call rate >90%) biallelic SNVs on the 38 canine autosomes were retained for the ROH analyses, which constituted a set of 6,344,366 SNVs. LD pruning was not applied before ROH detection according to previously published guidelines [32].

Based on mean observed heterozygosity, number of samples, and number of markers, a minimum number of SNVs used to identify a ROH was calculated to be 72 in order to produce less than 5% of randomly generated ROH according to the previously described method [33]. Parameters for the number of heterozygous calls in a window (1, 3, 5) and minimum kb size of ROH (100, 300, 1000) were varied and evaluated in order to avoid falsely breaking a long ROH [34]. Finally, three heterozygous calls per window were allowed, and a minimum length of 300 kb was required. Other ROH parameters were kept at the default value as defined in PLINK v1.9 [30]. The inbreeding coefficient based on ROH (F_ROH) was calculated for each dog as the total length of all its ROH divided by the total length of the autosomal genome covered by SNV positions (herein 2,225,350,880 bp). R v4.2.1 [29] was used to visualize all results and to compute one-way analysis of variance used to check statistical significance of mean differences between countries.

A threshold of 90% was set for the detection of ROH islands, meaning genomic regions without a minimum size requirement that were called with consecutive homozygous SNVs in at least 90% of the dogs. Such areas may indicate regions under selection or resulting from population bottlenecks. However, the presence of ROH islands does not implicate an identical underlying haplotype [33]. Gene content in the detected regions was investigated based on NCBI Annotation Release 106 of the canine genome assembly (GCF_011100685.1). The list of genes was manually inspected to identify genes with a previously described association with a specific trait or disease, which could be relevant to historical selection during the breed’s establishment. Additionally, the Online Mendelian Inheritance in Animals (OMIA) database was searched for previously described variants potentially relevant for Bernese mountain dogs, including alleles associated with three health disorders and four morphological traits, and their frequency was evaluated in the studied population. The individual risk index for histiocytic sarcoma was calculated for each dog based on the risk alleles described previously [8]. Based on this study, the index has three possible categories (A, B, or C). The C result corresponds to the greatest risk of developing histiocytic sarcoma, according to the Antagene laboratory (www.antagene.com, accessed on 20 January 2023) [34].

## 3. Results

### 3.1. Population Structure

**Figure 1:**
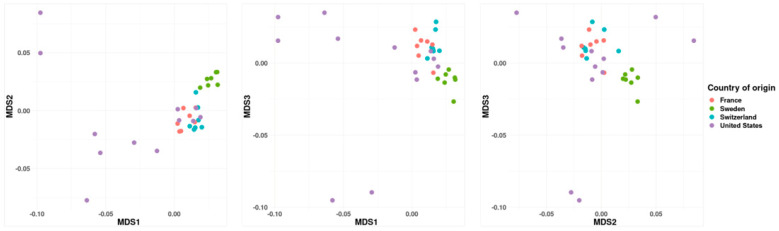
MDS analysis of the 33 dogs from four countries based on the first (MDS1), second (MDS2), and third (MDS3) dimensions using the LD-pruned variants dataset.

In total, 33 Bernese mountain dogs (13 males, 20 females) from four different countries were studied. The year of birth ranged from 1991 to 2019 (Supplementary Table S1). The MDS of pairwise genetic distances showed that the 21 European dogs clustered more closely to one another than to the 12 dogs from the USA (Figure 1), which were themselves more distributed than either of the other groups. There was evidence for regular import of breeding stock between the two continents, as about half of the USA dogs overlap the European group. The French and Swiss groups showed the largest overlap with each other, while the Swedish dogs formed a distinct cluster loosely separated from the other European dogs.

### 3.2. Runs of Homozygosity

ROH detection was performed on the dataset of 6,344,366 quality pruned SNVs. A total of 12,643 ROH were detected across all dogs, with a mean length of 2.29 Mb (ranging from 0.30 to 51.39 Mb). On average, 383 ROH were seen per individual with a range from 320 to 451. The total length of ROH across the genome per individual was 878.1 Mb, on average, and ranged from 704.7 to 1163.6 Mb (Supplementary Table S1). The average observed autosomal heterozygosity was 0.268.

**Figure 2:**
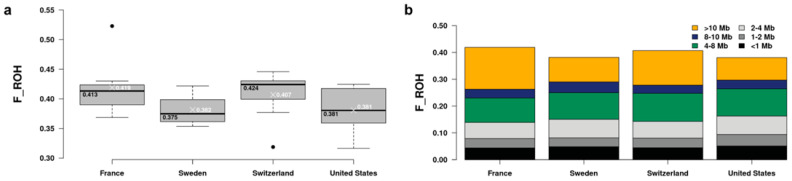
Comparison of the genomic inbreeding coefficients (F_ROH) between Bernese mountain dogs from the four countries. (**a**) Box plots of the F_ROH distribution in the European countries and the United States showing the median (black lines) and mean (white crosses) values in each group. (**b**) Average F_ROH per country; different colors represent contribution of the ROH length classes.

The autosomal ROH-based average genomic inbreeding coefficient (F_ROH) was 0.395 with a minimum value of 0.317 and a maximum of 0.523 (Supplementary Table S1). F_ROH varied slightly between countries. The highest median (0.424) was observed in Swiss dogs while the highest mean (0.419) was found in French dogs. The average F_ROH of the dogs from Sweden and the United States was about 3% lower (Figure 2a). However, these differences were not statistically significant (*p* > 0.05). ROH were further divided into six groups based on their length: <1, 1–2, 2–4, 4–8, 8–10, and >10 Mb. The relative contribution of these six ROH size classes to the average F_ROH per country is indicated in Figure 2b.

**Figure 3:**
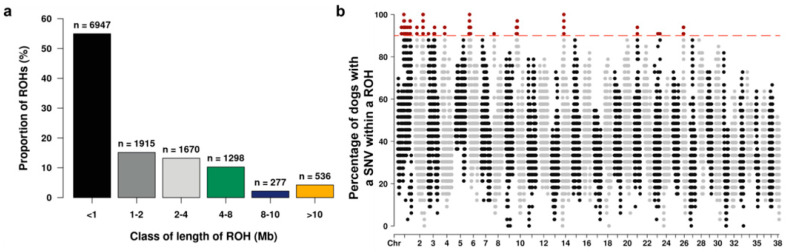
Genome-wide ROH analysis of the Bernese mountain dogs. (**a**) Distribution of six different ROH length classes. (**b**) Incidence plot showing the proportion of the 33 individuals with a specific SNV in a particular ROH across the 38 canine autosomes; red dashed line indicates the 90% cutoff for determination of the ROH islands (in dark red).

Overall, most of the detected ROH (83.3%) were shorter than 4 Mb, and 4.2% were longer than 10 Mb (Figure 3a). The number of ROH per chromosome ranged from 96 on chromosome 35 to 680 on chromosome 1; the proportion of homozygous regions relative to chromosomal size was highest for chromosome 35 (12.5%) and lowest for chromosome 4 (2.3%). The fraction of dogs having a specific SNV inside a ROH varied across the genome, with regions on 11 chromosomes exceeding the 90% incidence cutoff for ROH islands (Figure 3b). All 38 autosomes are shown in detail in Supplementary Figure S1.

### 3.3. ROH Islands

Of the 6,344,366 SNVs in the pruned dataset, 6,340,396 (99.9%) were located in a ROH in at least one dog. Of those, 48,841 (0.8%) SNVs on 11 chromosomes exceeded the 90% threshold among the 33 dogs assayed (Figure 3b). These SNVs formed 56 ROH islands on 11 chromosomes, in which 712 genes were annotated (Supplementary Table S2). Fourteen of these genes have a phenotype association described in different species in OMIA database, and 80 genes are associated with human phenotypes in the Online Inheritance in Man (OMIM) database (Supplementary Table S2). Notably, one of the detected islands is on chromosome 10 between 7.77 and 8.62 Mb and harbors 11 genes, including *MSRB3*, which has been previously associated with ear type in dogs (OMIA 000319-9615) [35]. Another 344.7 kb region on chromosome 21 showed shared homozygosity in 90.91% (30/33) of the individuals. In this ROH, 10 out of 17 annotated genes were olfactory receptors. By comparing the regions with those reported by Gorssen et al. [33], six chromosomes harbored segments that overlap between their results and the current study (Supplementary Table S2). The ear type-associated region was found by both methods and could be used as an example of a positive control.

Five genome regions on chr1, chr2, chr6, and chr14 were identified for which 100% of the dogs shared overlapping ROH (Table 1). These ROH islands were investigated visually in more detail to determine if all individuals had the same underlying haplotype. This was the case for all five regions. However, the analysis revealed five dogs that had two heterozygous stretches (~100 kb and ~70 kb) within the chr1 island, breaking it in two smaller regions: 55,492,829–55,583,781 and 55,662,185–55,698,676. Several of the genes annotated in these fixed homozygous regions are involved in inflammation and the regulation of immune cells (*CCR6*, *GPR31*, *SMPDL3B*, *THEMIS2*) or in DNA damage repair (*RPA2*, *PP1R8*) [36].

### 3.4. Distribution of Known Trait-Associated Variants

The frequency of previously described variants that are associated with specific traits in Bernese mountain dogs was also investigated. The findings represent eight disease-related [6,8,37,38,39] and eight morphological trait-related variants [40,41,42,43] (Table 2). In addition, 74 SNVs reported by Hédan et al. [8] were used to determine the four risk haplotypes for histiocytic sarcoma on three chromosomes. As shown in Table 2, the alternative allele frequency at these variants varied from 0 to 100%. For example, the *GFAP* variant associated with Alexander disease, which is characterized by myelin loss and abnormal protein deposits, was not detected at all, and all studied dogs had a homozygous reference genotype. In contrast, all three genotypes were found for the variants in *SOD1* and its modifier *SP110*, which are associated with degenerative myelopathy. All four risk haplotypes that have been described for development of histiocytic sarcoma in Bernese mountain dogs [8] were detected in the tested population with frequencies ranging from 62.1% to 72.7% (Table 2). The individual risk index was calculated for each dog, and its distribution in the 33 dogs (A = 0.21, B = 0.55, C = 0.24; Supplementary Table S1) was close to that described in the whole population reported by the Antagene laboratory (6700 tests: A = 0.26, B = 0.44, C = 0.30; [34]). Only the haplotype frequency of the chr11:41Mb locus differed significantly between European and USA dogs (European: 0.833 vs. USA: 0.542, *p* < 0.01), while the haplotype frequency at the other three loci was not significantly different (chr5: European and USA: 0.667; chr11:44Mb: European: 0.690 vs. USA: 0.458, *p* > 0.05; chr14: European: 0.547 vs. USA: 0.750, *p* > 0.05).

Variants associated with height, ear type, hair length, and coat color were also analyzed (Table 2). All but two of the dogs were homozygous for the T allele in the *IGF1* gene that was previously associated with large breeds (body mass > 25 kg) [41]. Similarly, 32 of 33 dogs were homozygous for a 10 bp deletion in the 3′-untranslated region of *MSRB3* reported previously in dogs with floppy ears [44]. All dogs were homozygous for the A allele in the *FGF5* gene commonly associated with long hair in various breeds [42]. No dog carried any of the four other rare *FGF5* long hair alleles described in Akita, Samoyed, Eurasier, and Afghan hound [43]. The distinct tricolor coat color of Bernese mountain dogs is considered to represent the ‘black back’ pattern caused by structural variants in two promoters of the *ASIP* gene [40]. All 33 Bernese mountain dogs in this study had two copies of the VP2-HCP3 extended haplotype, which has been described in dogs with the black back pattern [40].

### 3.5. Mitochondrial Diversity

Only two mitochondrial haplotypes were detected in the 33 sampled dogs, and both belong to the haplogroup A. Following the nomenclature standard based on the mtDNA D-loop region [25], seven dogs carried the A2 haplotype and 26 had the A22 haplotype. When considering the complete mtDNA standardized classification system [26], the seven dogs belonged to haplotype A1b2a1a1 and the 26 dogs to the A1b3b2 haplotype. The less frequent haplotype was present in individuals from all countries except Switzerland (Supplementary Table S3). In addition, two synonymous SNVs in the coding region of the mitogenome were detected: a variant in the *ND5* gene (CM022001.1: m.4378A>G) appeared in three Swedish and one USA dog, and a variant in the *ATP6* gene (CM022001.1: m.7802A>G) was observed in a single French dog.

## 4. Discussion

This study of Bernese mountain dogs from Europe and the United States demonstrated that breed genetic diversity is limited, as would be expected in a typical modern dog breed [10]. The observed average inbreeding coefficient of 0.395 detected herein is comparable to that reported in previous studies in Bernese mountain dogs, e.g., Dreger et al. reported an inbreeding coefficient of 0.350 using SNP genotyping data from ten dogs and 0.314 using WGS data from two dogs [45]; Bannasch et al. estimated an inbreeding coefficient of 0.317 based on SNP array data from 306 dogs [46]. The detected value is high, although not surprising in an inbred dog breed, and falls within the range reported in other breeds, such as Greater Swiss mountain dog (0.426), Boxer (0.395), or Dobermann (0.388) [46]. However, other common breeds show lower values, such as Labrador retriever (0.218) [46], German shorthaired pointer (0.173) [11], or Border collie (0.037) [14]. By comparison, Norwegian Lundehund is an example of an extremely inbred domestic breed predisposed to a multifactorial life-threatening Lundehund syndrome with a reported inbreeding coefficient as high as 0.87 [16,47].

In total, 12,643 ROH were detected across all dogs, with an average length of 2.29 Mb. Generally, shorter ROH are considered to be indicators of more distant relatedness, while longer segments are a result of more recent inbreeding [48]. Herein, more than 80% of ROH were shorter than 4 Mb, and only about 4% represented segments longer than 10 Mb. In contrast, in the Leonberger breed, it has been reported that ~54% of ROH are shorter than 4 MB and ~16% are longer than 10 Mb, suggesting both historical bottlenecks and frequent recent inbreeding practices [17]. It can therefore be assumed that the high relatedness in Bernese mountain dogs is likely a result of population founder effects and historical selection for particular traits but also a relatively recent increase in inbreeding due to overuse of popular sires. Such breeding practices have been documented to increase the likelihood of developing certain diseases, such as histiocytic sarcoma, and to decrease the average lifespan [9]. While not statistically significant, small differences were seen between dogs from the studied countries, potentially indicating different breeding practices (more recent and/or intensive inbreeding) in the Swiss and French breed clubs in comparison to the Swedish or USA populations. The MDS of genetic distances suggested regular exchange of dogs for different breeding programs between countries and continents, as was also documented before [9,49].

In a previous study, Gorssen et al. [33] reported 26 ROH islands on nine chromosomes in Bernese mountain dogs using medium-density 170 k SNP genotyping data of 34 dogs. Here, we detected 56 ROH islands on 11 chromosomes using WGS of 33 dogs. Even though different methods were utilized, we observed six chromosomes that harbor segments, which overlap between the two datasets. For example, the *MSRB3* region on chromosome 10, which has been associated with body mass and ear type, was found in both studies, highlighting the value of public repositories of data that can serve as references for other researchers and the value of validation studies. These regions have likely been under selection by breeders and should be investigated further in future studies.

Selected variants that may play a role in Bernese mountain dog health were genotyped in the 33 purebred dogs with no report of clinical signs of disease at the time of sampling. Two autosomal recessive *SOD1* alleles with incomplete penetrance have been reported in dogs affected by degenerative myelopathy, an adult-onset progressive neurological disease [6,37]. The first allele is common among breeds, while the second is rare and is, thus far, limited to Bernese mountain dogs. In our dataset, both alleles were present, with allele frequencies of 0.379 and 0.015, in accordance with previous reports [50]. Alexander disease is a rare progressive neurological disorder with young age of onset and was described previously in multiple breeds, including Bernese mountain dog. However, only one causal variant for an autosomal dominant allele has been found to date in Labrador retrievers [39]. That variant was absent in the 33 analyzed Bernese mountain dogs. This is perhaps not surprising, as the disease is rare in both humans and dogs, although there was a case report in a Bernese mountain dog [51]. The risk haplotype frequencies of the four loci identified in histiocytic sarcoma were intermediate to those observed in the affected and unaffected dogs of the original study by Hédan et al. [8], which is in agreement with the fact that the studied dogs are representative of the whole population and were reported unaffected at the time of sampling. Significant difference between the frequency of risk alleles on chr 11 were observed between the European and USA dogs, which is concordant with a previous publication by Shearin et al. [52].

In the context of strong breed predisposition to many hematopoietic cancers, especially histiocytic sarcomas, involving dendritic cells or macrophages, it could be suspected that Bernese mountain dogs present fixed homozygous regions related to immune dysregulation [8]. Five ROH islands homozygous in all 33 dogs were found harboring 32 annotated genes. Notably, *CCR6* encodes a chemokine receptor that has been involved in inflammatory and infectious diseases and that is associated with several cancers through immune cell control [53], while *SMPDL3B* is involved in innate immunity and may be used as a prognostic biomarker in human cancers, such as myeloid leukemia and ductal carcinoma [54,55], and *THEMIS2* is expressed in a variety of cancer types and cell lines and was recently found to have tumor-suppressive role in breast cancer [56].

The fixed alleles associated with long hair and coat color typical for the breed were not detected as ROH islands. Complex structural variation such as in the *ASIP* promoter poses a technical problem due to many erroneous heterozygous calls in such regions. Furthermore, due to the extensive selection in the breed’s history, fixed haplotypes may have become too small to detect using the selected parameters. This is the most likely explanation for the *FGF5* locus in Bernese mountain dogs, in which the size of the shared homozygous region was only ~2.4 kb. The fact that two variants with influence on body mass and ear type (*IGF1* and *MSRB3*) were not fixed in all studied Bernese mountain dogs, even though the breed is homogeneous in these morphological characteristics, supports the notion that additional variants, perhaps in other genes, remain to be found [57].

Two mtDNA haplotypes, A1b2a1a1 (A2) and A1b3b2 (A22), were detected in this study, which correspond to previous reports in Bernese mountain dogs [25,26,58,59]. The A haplogroup is the most frequent among dog breeds, and these specific haplotypes are widespread in a number of breeds; namely, haplotype A2 was detected in more than 20 diverse breeds such as Borzoi, Great Dane, or Shih Tzu, and haplotype A22 in at least 14 breeds including Irish wolfhound, Saint Bernard, or Tibetan terrier [25,26,58,59].

## 5. Conclusions

Whole-genome sequence data from 33 Bernese mountain dogs sampled from four countries demonstrated that within-breed diversity is low, likely due to artificial selection throughout the breed’s history, leading to high levels of inbreeding and relatedness in the current population. The detection of common ROH islands may facilitate further studies to clarify the functional impact of genes involved in these highly homozygous regions. Whole-genome analysis is efficient for the detection of breed-related variants and regions under selection and may be used for individual disease risk assessment and differential follow up of the animal’s health status. Such breed population genomic studies will enable breeders and kennel clubs to evaluate and set relevant rules for the monitoring and management of highly prevalent genetic disorders that severely affect the dog’s quality of life and diminish the breed’s average lifespan. Preserving the current genetic diversity level of Bernese mountain dog would be possible by increasing the overall number of sires in breeding programs while limiting the number of litters per sire/dam and avoiding repeated matings. In addition, genetic testing for known recessive diseases can be performed to help avoid carrier pairing. The use of HS risk test over almost 10 years showed that there is enough diversity for breeders to slowly select against cancer by favoring the dogs with index A. However, in light of the complexity of many disorders prevalent in this breed, outcrossing may be an effective breeding strategy for long-term genetic diversity improvement, which would contribute to population sustainability and health.

## Figures and Tables

**Table 1:** List of detected ROH islands with 100% incidence and their gene content.

Chromosome	Region		Genes Annotated in the Region
	Start	End	
1	55,492,829	55,778,313	CCR6, GPR31, LOC484082, LOC111095971, TTLL2 *, LOC111095973 *, UNC93A *, LOC100855528 *, LOC102153253, LOC111090695 *, LOC111095985 *
2	71,838,359	71,946,097	*SMPDL3B, RPA2, THEMIS2, LOC119870383, PPP1R8, LOC119870569*
2	73,728,360	74,031,681	*LDLRAP1, LOC119870404, MACO1, RHCE, TMEM50A, RSRP1, LOC478183, LOC478184, LOC119870544*
6	5,429,019	5,573,855	*CASTOR2, RCC1L, LOC100682844, LOC119872393*
14	15,015,814	15,089,249	*ZNF804B, LOC102153442*

* These six genes were in an area of continuous heterozygous genotypes in five animals.

**Table 2:** List of selected breed-relevant variants and their distribution in 33 Bernese mountain dogs.

Trait/ Disorder	OMIA ID	Gene	Variant Region ^1^	AlternativeAllele/ Haplotype Frequency	Counts		
					Ref/Ref	Ref/Alt	Alt/Alt
Degenerative myelopathy	000263-9615	*SOD1*	chr31:g.27123057G>A	0.379	10	21	2
			chr31:g.27118886A>T	0.015	32	1	0
		*SP110*	chr25:g.42620442G>A	0.652	4	15	14
			chr25:g.42623004C>T	0.636	5	14	14
			chr25:g.42628769C>T	0.167	23	9	1
			chr25:g.42631341G>A	0.167	23	9	1
			chr25:g.42633078T>A	0.348	14	15	4
Alexander disease	001208-9615	*GFAP*	chr9:g.18459694G>A	0.000	33	0	0
Histiocytic sarcoma	000620-9615	risk haplotype	chr5:33Mb ^2^	0.667	5	12	16
		risk haplotype	chr11:41Mb ^3^	0.727	2	14	17
		risk haplotype	chr11:44Mb ^4^	0.621	4	17	12
		risk haplotype	chr14:11Mb ^5^	0.621	3	19	11
Height	002524-9615	*IGF1*	chr15:g.41511739T>C	0.030	31	2	0
Ear type	000319-9615	*MSRB3*	chr10:g.8395407delTTTATTTTAT	0.970	1	0	32
Hair length	000439-9615	*FGF5*	chr32:g.35475211G>A	0.000	33	0	0
			chr32:g.35475230_35475231dup	0.000	33	0	0
			chr32:g.35475218_35475233del	0.000	33	0	0
			chr32:g.35486609A>T	0.000	33	0	0
			chr32:g.35494497C>A	1.000	0	0	33
Coat color	000601-9615	*ASIP*	chr24:g.[23863804_23863988del; 23865233_23865267A [4]; 23891659_23891781delins [MT319115.1:424_674]	1.000	0	0	33

^1^ All positions refer to the dog reference genome assembly UU_Cfam_GSD_1.0. ^2^ The haplotype was determined by the genotypes of the following SNVs: 33855429, 33993463, 34005879, 34012145, 34028329, 34043717, 34076942, 34083675, 34087262, 34087720, 34100829, 34103314, 34109473, 34120114, 34421555, 34425934, 34428714, 34433959, 34434313, 34436053, 34440306, 34440578, 34442862, 34448188, 34449394, 34455245, 34529929.^3^ The haplotype was determined by the genotypes of the following SNVs: 41258181, 41275129, 41275213, 41277330, 41280608, 41290542, 41298930, 41306401, 41310312, 41313739, 41317800, 41329384, 41330760, 41332109, 41366494. ^4^ The haplotype was determined by the genotypes of the following SNVs: 43510563, 43519952, 43537944, 43565114, 43939450, 44316022, 44342776, 44343823, 44344884, 44366118, 44371942, 44374045, 44380626, 44395661, 44397515, 44609562, 44611627, 44612055. ^5^ The haplotype was determined by the genotypes of the following SNVs: 10887016, 10889217, 10891570, 10893694, 10895560, 10898604, 10906919, 10932956, 10936449, 10943176, 10949045, 10958187, 10960590, 10974467.

## Data Availability

The genome sequence data used in this study are available from the European Nucleotide Archive. Accession numbers are given in Supplementary Table S1.

## Supplementary Materials

The following supporting information can be downloaded at: https://www.mdpi.com/article/10.3390/genes14030650/s1, Table S1: Information about the studied genomes of 33 Bernese mountain dogs; Table S2: List of detected runs of homozygosity islands including the annotated genes in those regions; Table S3: Diagnostic positions in mtDNA used for detection of haplotypes in Bernese mountain dogs; Figure S1: Proportion of dogs with a SNP within a run of homozygosity on each of the 38 autosomes.

## References

[1] Fédération Cynologique Internationale. http://www.fci.be/en/.

[2] Räber H. (2008). Die Schweizer Hunderassen.

[3] Nielsen L., Andreasen S.N., Andersen S.D., Kristensen A.T. (2010). Malignant Histiocytosis and Other Causes of Death in Bernese Mountain Dogs in Denmark. Vet. Rec..

[4] Klopfenstein M., Howard J., Rossetti M., Geissbühler U. (2016). Life Expectancy and Causes of Death in Bernese Mountain Dogs in Switzerland. BMC Vet. Res..

[5] Kathmann I., Jaggy A., Busato A., Bärtschi M., Gaillard C. (1999). Clinical and Genetic Investigations of Idiopathic Epilepsy in the Bernese Mountain Dog. J. Small Anim. Pract..

[6] Wininger F.A., Zeng R., Johnson G.S., Katz M.L., Johnson G.C., Bush W.W., Jarboe J.M., Coates J.R. (2011). Degenerative Myelopathy in a Bernese Mountain Dog with a Novel SOD1 Missense Mutation. J. Vet. Intern. Med..

[7] Ohlerth S., Geiser B., Flückiger M., Geissbühler U. (2019). Prevalence of Canine Hip Dysplasia in Switzerland Between 1995 and 2016—A Retrospective Study in 5 Common Large Breeds. Front. Vet. Sci..

[8] Hédan B., Cadieu É., Rimbault M., Vaysse A., de Citres C.D., Devauchelle P., Botherel N., Abadie J., Quignon P., Derrien T. (2021). Identification of Common Predisposing Loci to Hematopoietic Cancers in Four Dog Breeds. PLoS Genet.

[9] Abadie J., Hédan B., Cadieu E., de Brito C., Devauchelle P., Bourgain C., Parker H.G., Vaysse A., Margaritte-Jeannin P., Galibert F. (2009). Epidemiology, Pathology, and Genetics of Histiocytic Sarcoma in the Bernese Mountain Dog Breed. J. Hered..

[10] Broeckx B.J.G. (2020). The Dog 2.0: Lessons Learned from the Past. Theriogenology.

[11] Boccardo A., Marelli S.P., Pravettoni D., Bagnato A., Busca G.A., Strillacci M.G. (2020). The German Shorthair Pointer Dog Breed (Canis Lupus Familiaris): Genomic Inbreeding and Variability. Animals.

[12] Mastrangelo S., Biscarini F., Tolone M., Auzino B., Ragatzu M., Spaterna A., Ciampolini R. (2018). Genomic Characterization of the Braque Français Type Pyrénées Dog and Relationship with Other Breeds. PLoS ONE.

[13] Mortlock S.A., Khatkar M.S., Williamson P. (2016). Comparative Analysis of Genome Diversity in Bullmastiff Dogs. PLoS ONE.

[14] Soh P.X.Y., Hsu W.T., Khatkar M.S., Williamson P. (2021). Evaluation of Genetic Diversity and Management of Disease in Border Collie Dogs. Sci. Rep..

[15] Friedenberg S.G., Meurs K.M., Mackay T.F.C. (2016). Evaluation of Artificial Selection in Standard Poodles Using Whole-Genome Sequencing. Mamm. Genome.

[16] Metzger J., Pfahler S., Distl O. (2016). Variant Detection and Runs of Homozygosity in next Generation Sequencing Data Elucidate the Genetic Background of Lundehund Syndrome. BMC Genom..

[17] Letko A., Minor K.M., Jagannathan V., Seefried F.R., Mickelson J.R., Oliehoek P., Drögemüller C. (2020). Genomic Diversity and Population Structure of the Leonberger Dog Breed. Genet. Sel. Evol..

[18] André C., Botherel N., Cadieu E., Lagoutte L., Hédan B., Garand A., Abadie J., Tiret L., Abitbol M., Lavoué R. (2022). Cani-DNA, Un CRB Qui a Du Chien ! Réseau de Collecte de Prélèvements de Chiens Par Les Vétérinaires Pour La Recherche Biomédicale et La Diversité Génétique. NOV’AE.

[19] Ostrander E.A., Wang G.-D., Larson G., VonHoldt B.M., Davis B.W., Jagannathan V., Hitte C., Wayne R.K., Zhang Y.-P., André C. (2019). Dog10K: An International Sequencing Effort to Advance Studies of Canine Domestication, Phenotypes and Health. Natl. Sci. Rev..

[20] Jagannathan V., Drögemüller C., Leeb T. (2019). Dog Biomedical Variant Database Consortium, (DBVDC) A Comprehensive Biomedical Variant Catalogue Based on Whole Genome Sequences of 582 Dogs and Eight Wolves. Anim. Genet..

[21] Chen S., Zhou Y., Chen Y., Gu J. (2018). Fastp: An Ultra-Fast All-in-One FASTQ Preprocessor. Bioinformatics.

[22] Wang C., Wallerman O., Arendt M.-L., Sundström E., Karlsson Å., Nordin J., Mäkeläinen S., Pielberg G.R., Hanson J., Ohlsson Å. (2021). A Novel Canine Reference Genome Resolves Genomic Architecture and Uncovers Transcript Complexity. Commun. Biol..

[23] Vasimuddin M., Misra S., Li H., Aluru S. Efficient Architecture-Aware Acceleration of BWA-MEM for Multicore Systems. Proceedings of the 2019 IEEE 33rd International Parallel and Distributed Processing Symposium, IPDPS 2019.

[24] Van der Auwera G.A., O’Connor B.D. (2020). Genomics in the Cloud: Using Docker, GATK, and WDL in Terra.

[25] Pereira L., Van Asch B., Amorim A. (2004). Standardisation of Nomenclature for Dog MtDNA D-Loop: A Prerequisite for Launching a Canis Familiaris Database. Forensic. Sci. Int..

[26] Duleba A., Skonieczna K., Bogdanowicz W., Malyarchuk B., Grzybowski T. (2015). Complete Mitochondrial Genome Database and Standardized Classification System for Canis Lupus Familiaris. Forensic. Sci. Int. Genet..

[27] Thorvaldsdóttir H., Robinson J.T., Mesirov J.P. (2013). Integrative Genomics Viewer (IGV): High-Performance Genomics Data Visualization and Exploration. Brief. Bioinform..

[28] Chang C.C., Chow C.C., Tellier L.C.A.M., Vattikuti S., Purcell S.M., Lee J.J. (2015). Second-Generation PLINK: Rising to the Challenge of Larger and Richer Datasets. Gigascience.

[29] R Core Team (2019). R: A Language and Environment for Statistical Computing.

[30] Meyermans R., Gorssen W., Buys N., Janssens S. (2020). How to Study Runs of Homozygosity Using Plink? A Guide for Analyzing Medium Density Snp Data in Livestock and Pet Species. BMC Genom..

[31] Lencz T., Lambert C., DeRosse P., Burdick K.E., Morgan T.V., Kane J.M., Kucherlapati R., Malhotra A.K. (2007). Runs of Homozygosity Reveal Highly Penetrant Recessive Loci in Schizophrenia. Proc. Natl. Acad. Sci. USA.

[32] Ceballos F.C., Joshi P.K., Clark D.W., Ramsay M., Wilson J.F. (2018). Runs of Homozygosity: Windows into Population History and Trait Architecture. Nat. Rev. Genet..

[33] Gorssen W., Meyermans R., Janssens S., Buys N. (2021). A Publicly Available Repository of ROH Islands Reveals Signatures of Selection in Different Livestock and Pet Species. Genet. Sel. Evol..

[34] Hédan B. Molecular Basis of Canine Histiocytic Sarcoma in Dogs. https://berner-iwg.org/wp-content/uploads/2022/10/6-Dr-Benoit-Hedan-IWG-2022.pdf.

[35] Boyko A.R., Quignon P., Li L., Schoenebeck J.J., Degenhardt J.D., Lohmueller K.E., Zhao K., Brisbin A., Parker H.G., vonHoldt B.M. (2010). A Simple Genetic Architecture Underlies Morphological Variation in Dogs. PLoS Biol..

[36] Gene. Bethesda (MD): National Library of Medicine (US), National Center for Biotechnology Information. https://www.ncbi.nlm.nih.gov/gene/.

[37] Awano T., Johnson G.S., Wade C.M., Katz M.L., Johnson G.C., Taylor J.F., Perloski M., Biagi T., Baranowska I., Long S. (2009). Genome-Wide Association Analysis Reveals a SOD1 Mutation in Canine Degenerative Myelopathy That Resemblesnamyotrophic Lateral Sclerosis. Proc. Natl. Acad. Sci. USA.

[38] Ivansson E.L., Megquier K., Kozyrev S.V., Murén E., Körberg I.B., Swofford R., Koltookian M., Tonomura N., Zeng R., Kolicheski A.L. (2016). Variants within the SP110 Nuclear Body Protein Modify Risk of Canine Degenerative Myelopathy. Proc. Natl. Acad. Sci. USA.

[39] van Poucke M., Martlé V., van Brantegem L., Ducatelle R., van Ham L., Bhatti S., Peelman L.J. (2015). A Canine Orthologue of the Human GFAP c.716G>A (p.Arg239His) Variant Causes Alexander Disease in a Labrador Retriever. Eur. J. Hum. Genet..

[40] Bannasch D.L., Kaelin C.B., Letko A., Loechel R., Hug P., Jagannathan V., Henkel J., Roosje P., Hytönen M.K., Lohi H. (2021). Dog Colour Patterns Explained by Modular Promoters of Ancient Canid Origin. Nat. Ecol. Evol..

[41] Plassais J., vonHoldt B.M., Parker H.G., Carmagnini A., Dubos N., Papa I., Bevant K., Derrien T., Hennelly L.M., Whitaker D.T. (2022). Natural and Human-Driven Selection of a Single Non-Coding Body Size Variant in Ancient and Modern Canids. Curr. Biol..

[42] Housley D.J.E., Venta P.J. (2006). The Long and the Short of It: Evidence That FGF5 Is a Major Determinant of Canine ‘Hair’-Itability. Anim. Genet..

[43] Dierks C., Mömke S., Philipp U., Distl O. (2013). Allelic Heterogeneity of FGF5 Mutations Causes the Long-Hair Phenotype in Dogs. Anim. Genet..

[44] Chew T., Willet C.E., Haase B., Wade C.M. (2019). Genomic Characterization of External Morphology Traits in Kelpies Does Not Support Common Ancestry with the Australian Dingo. Genes.

[45] Dreger D.L., Rimbault M., Davis B.W., Bhatnagar A., Parker H.G., Ostrander E.A. (2016). Whole Genome Sequence, SNP Chips and Pedigree Structure: Building Demographic Profiles in Domestic Dog Breeds to Optimize Genetic Trait Mapping. Dis. Model Mech..

[46] Bannasch D., Famula T., Donner J., Anderson H., Honkanen L., Batcher K., Safra N., Thomasy S., Rebhun R. (2021). The Effect of Inbreeding, Body Size and Morphology on Health in Dog Breeds. Canine Med. Genet..

[47] Pfahler S., Distl O. (2014). A Massive Reduction of the Genetic Diversity in the Lundehund. Anim. Genet..

[48] Curik I., Ferenčaković M., Sölkner J. (2014). Inbreeding and Runs of Homozygosity: A Possible Solution to an Old Problem. Livest. Sci..

[49] Quignon P., Herbin L., Cadieu E., Kirkness E.F., Hédan B., Mosher D.S., Galibert F., André C., Ostrander E.A., Hitte C. (2007). Canine Population Structure: Assessment and Impact of Intra-Breed Stratification on SNP-Based Association Studies. PLoS ONE.

[50] Zeng R., Coates J.R., Johnson G.C., Hansen L., Awano T., Kolicheski A., Ivansson E., Perloski M., Lindblad-Toh K., O’Brien D.P. (2014). Breed Distribution of SOD1 Alleles Previously Associated with Canine Degenerative Myelopathy. J. Vet. Intern. Med..

[51] Wrzosek M., Giza E., Plonek M., Podgórski P., Vandevelde M. (2015). Alexander Disease in a Dog: Case Presentation of Electrodiagnostic, Magnetic Resonance Imaging and Histopathologic Findings with Review of Literature. BMC Vet. Res..

[52] Shearin A.L., Hédan B., Cadieu E., Erich S.A., Schmidt E.V., Faden D.L., Cullen J., Abadie J., Kwon E.M., Gröne A. (2012). The *MTAP-CDKN2A* Locus Confers Susceptibility to a Naturally Occurring Canine Cancer. Cancer Epidemiol. Biomark. Prev..

[53] Kadomoto S., Izumi K., Mizokami A. (2020). The CCL20-CCR6 Axis in Cancer Progression. Int. J. Mol. Sci..

[54] Kim S.J., Lee J.H., Park W.J., Kim S. (2021). Bioinformatic Exploration for Prognostic Significance of Sphingolipid Metabolism-Related Genes in Invasive Ductal Carcinoma Using the Cancer Genome Atlas Cohort. Int. J. Gen. Med..

[55] Qu H., Zhu Y. (2021). SMPDL3B Predicts Poor Prognosis and Contributes to Development of Acute Myeloid Leukemia. Front. Mol. Biosci..

[56] Huang W.C., Yen J.H., Sung Y.W., Tung S.L., Chen P.M., Chu P.Y., Shih Y.C., Chi H.C., Huang Y.C., Huang S.J. (2022). Novel Function of THEMIS2 in the Enhancement of Cancer Stemness and Chemoresistance by Releasing PTP1B from MET. Oncogene.

[57] Plassais J., Kim J., Davis B.W., Karyadi D.M., Hogan A.N., Harris A.C., Decker B., Parker H.G., Ostrander E.A. (2019). Whole Genome Sequencing of Canids Reveals Genomic Regions under Selection and Variants Influencing Morphology. Nat. Commun..

[58] Imes D.L., Wictum E.J., Allard M.W., Sacks B.N. (2012). Identification of Single Nucleotide Polymorphisms within the MtDNA Genome of the Domestic Dog to Discriminate Individuals with Common HVI Haplotypes. Forensic. Sci. Int. Genet..

[59] Verscheure S., Backeljau T., Desmyter S. (2014). Dog Mitochondrial Genome Sequencing to Enhance Dog MtDNA Discrimination Power in Forensic Casework. Forensic. Sci. Int. Genet..

