# Supplementary material for: Genomic Diversity and Runs of Homozygosity in Bernese Mountain Dogs
Source: Genes (Basel). 2023 Mar 4;14(3):650. doi: 10.3390/genes14030650 (PMC10048372; doi:10.3390/genes14030650)
Supplement: Supplementary file 1 [file genes-14-00650-s001.zip › FigureS1.pdf]

Supplementary Figure S1. Proportion of dogs with a SNP within a ROH on each of the 38 autosomes.

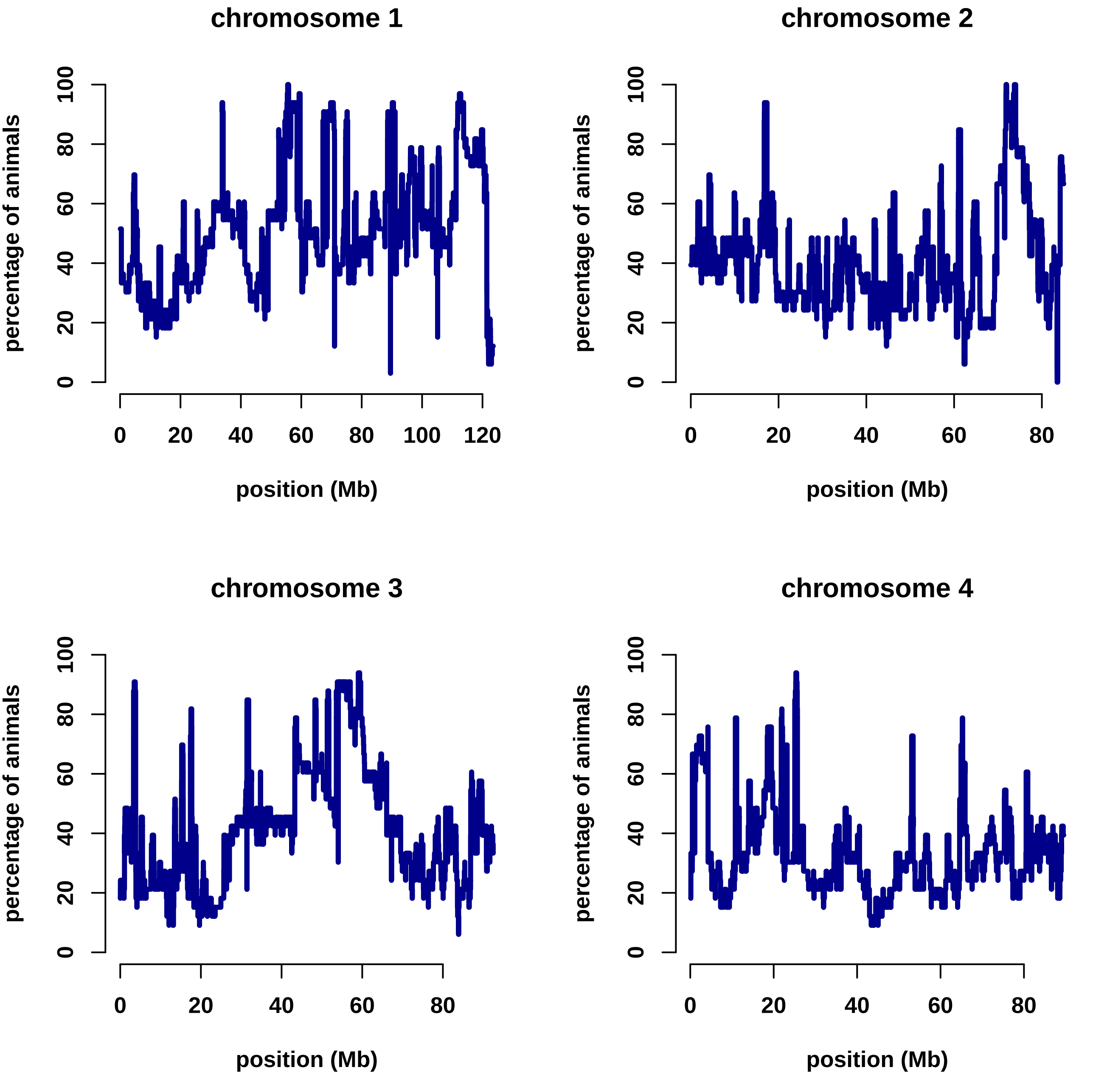

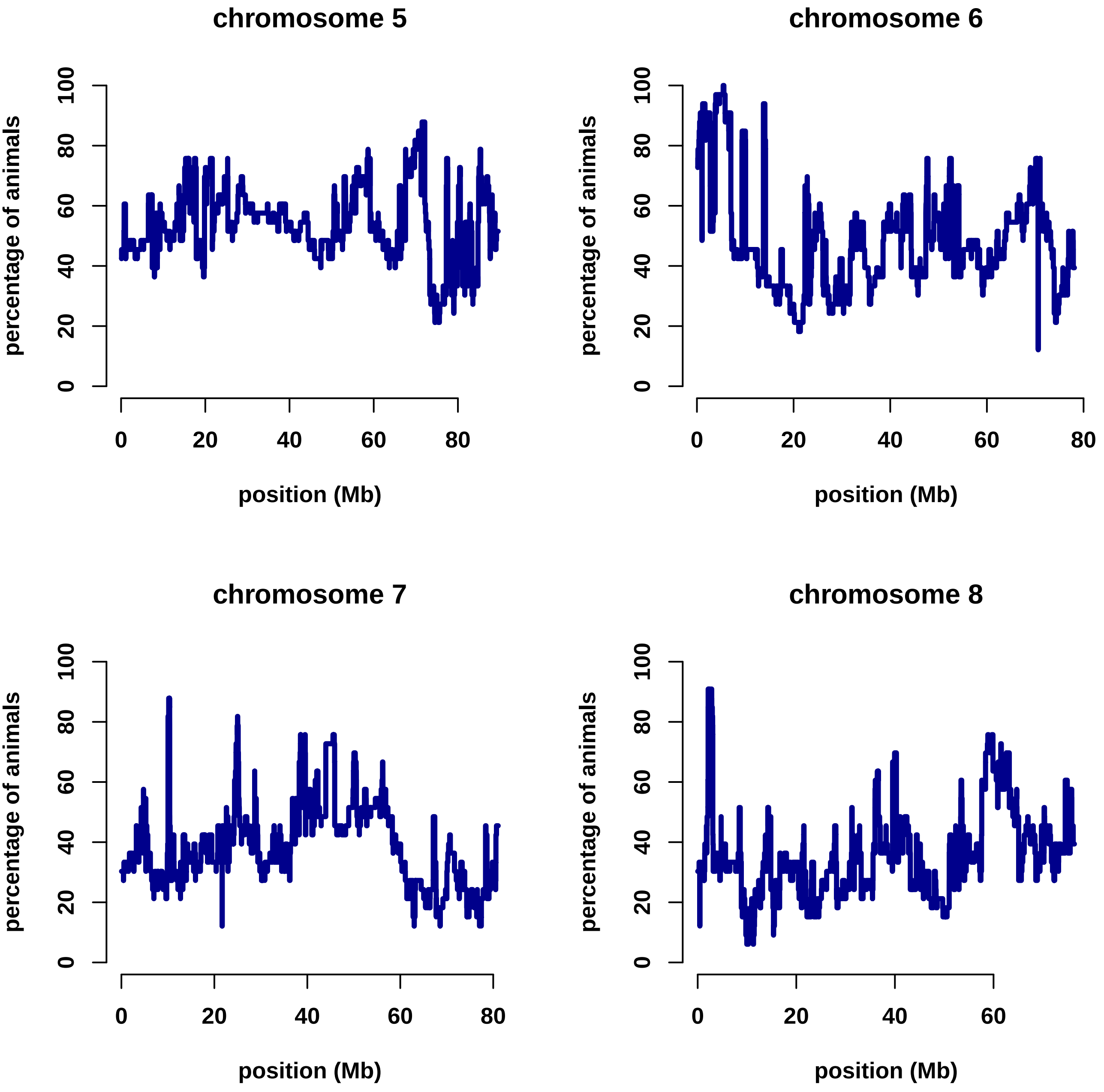
chromosome 5
chromosome 6
chromosome 7
chromosome 8
percentage of animals
position (Mb)
0
20
40
60
80
100

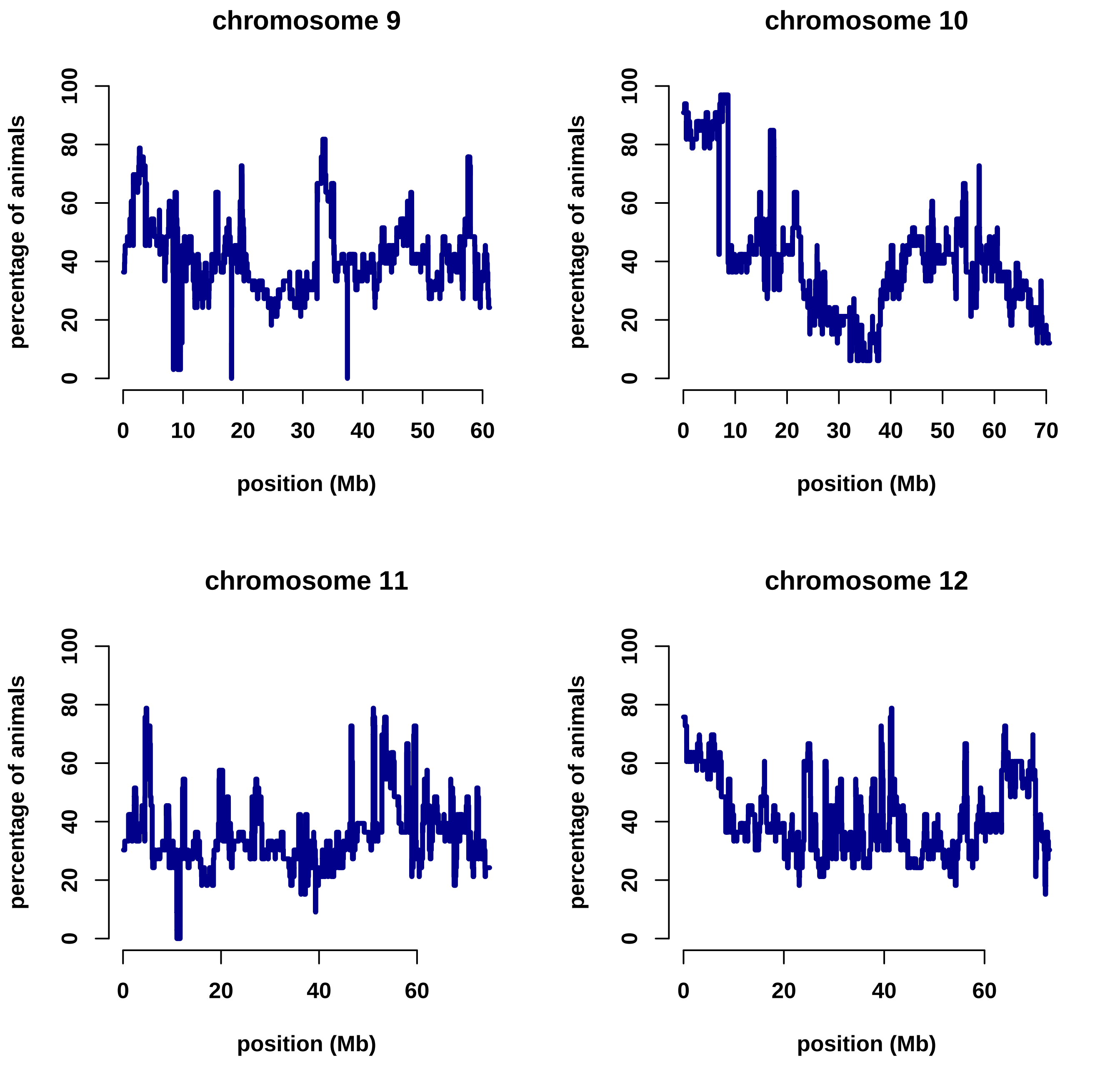
chromosome 9
chromosome 10
chromosome 11
chromosome 12
percentage of animals
0
20
40
60
80
100
position (Mb)
0
10
20
30
40
50
60
70

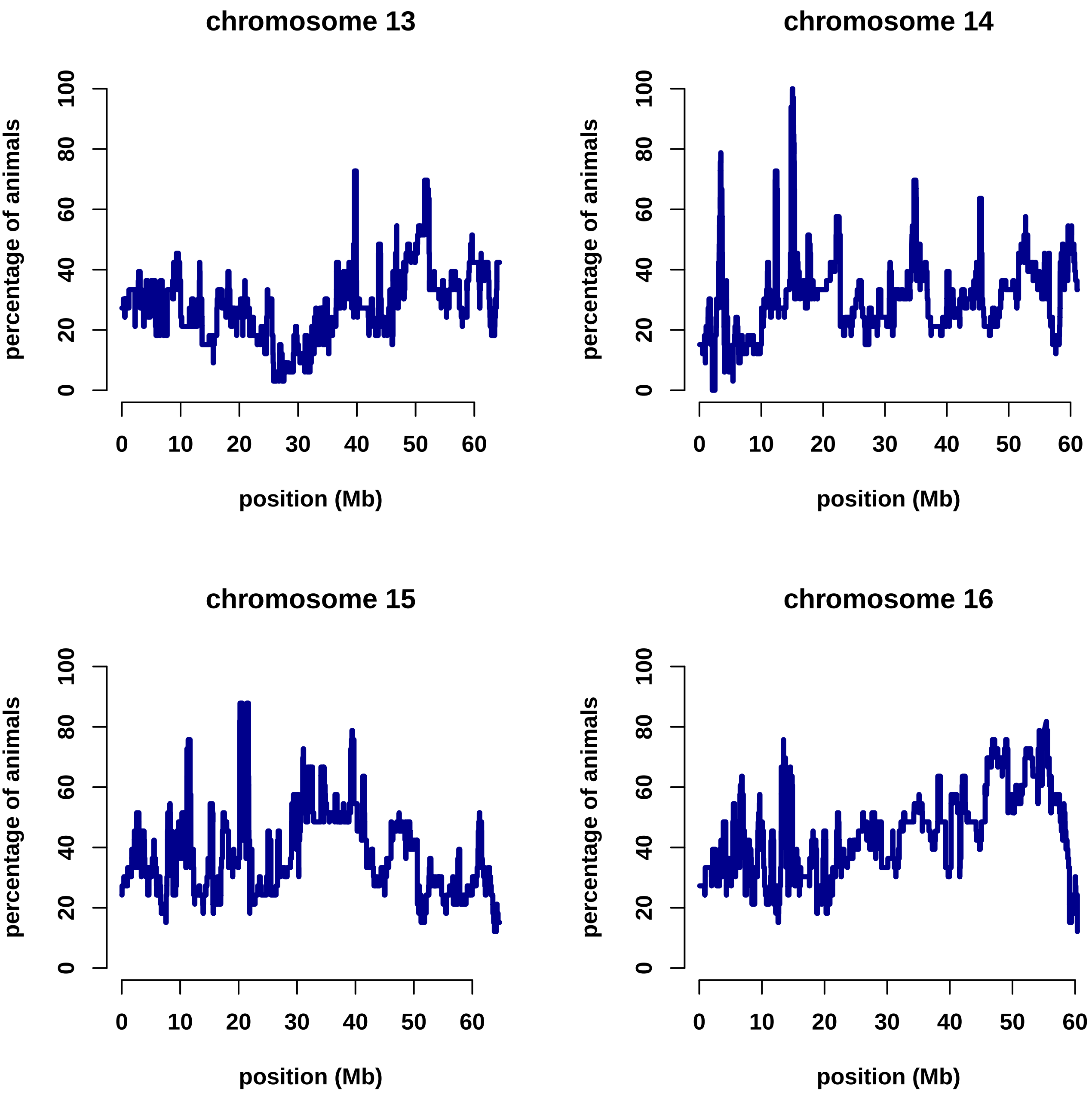
chromosome 13
chromosome 14
chromosome 15
chromosome 16
percentage of animals
position (Mb)
0
20
40
60
80
100
0
10
20
30
40
50
60

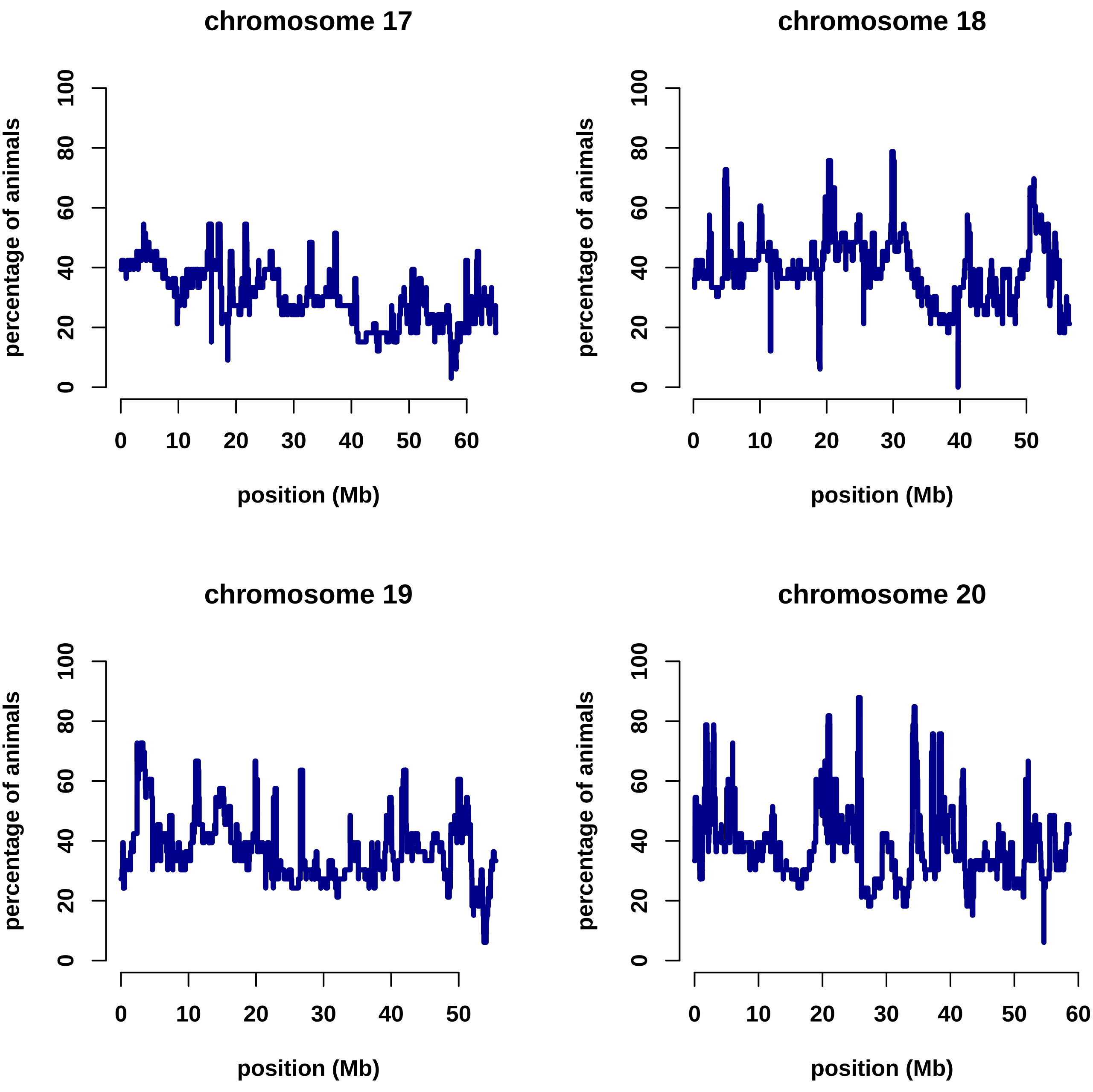
chromosome 17
chromosome 18
chromosome 19
chromosome 20
percentage of animals
0
20
40
60
80
100
position (Mb)
0
10
20
30
40
50
60

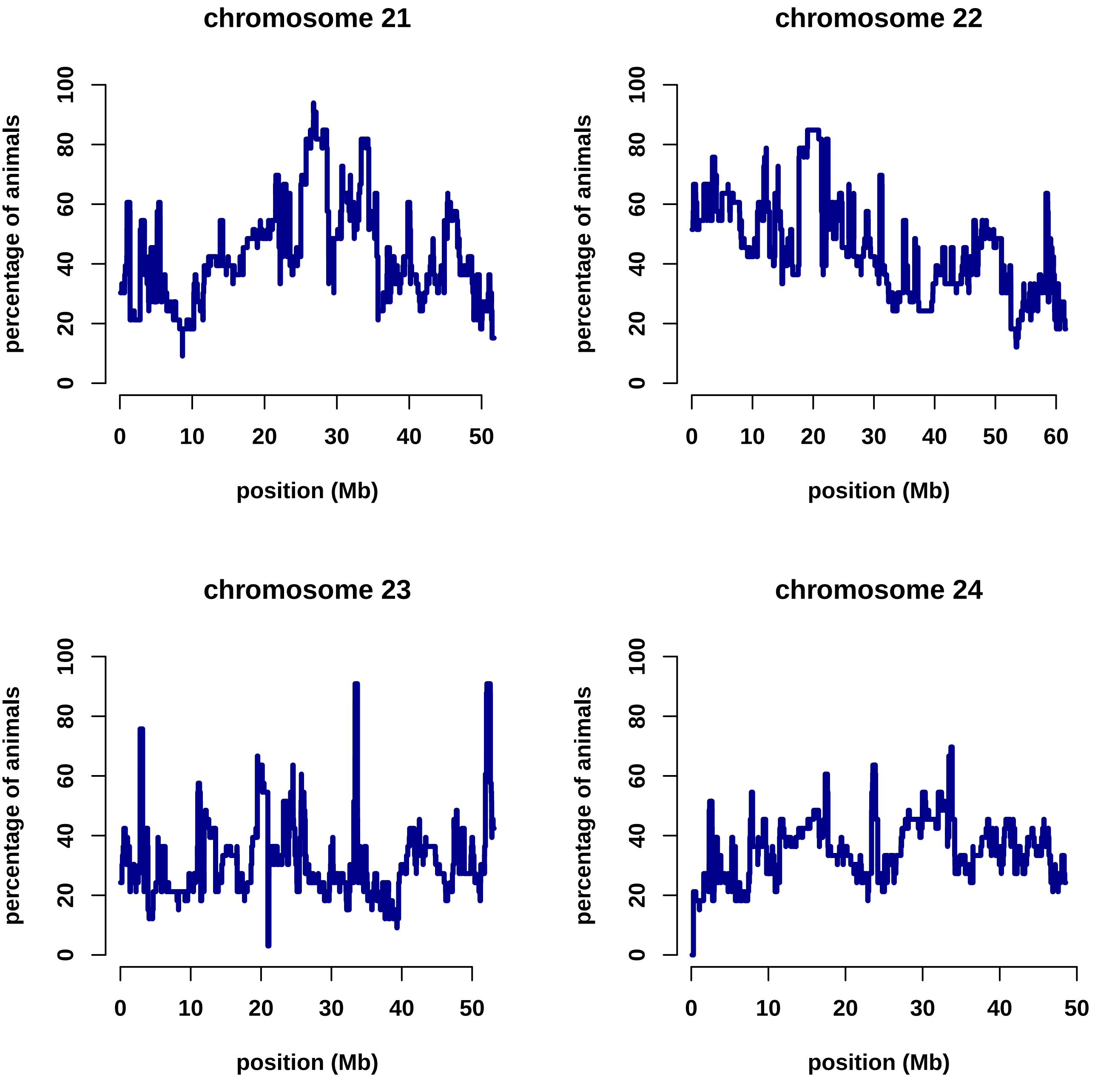
chromosome 21
chromosome 22
chromosome 23
chromosome 24
percentage of animals
0
20
40
60
80
100
position (Mb)
0
10
20
30
40
50
60

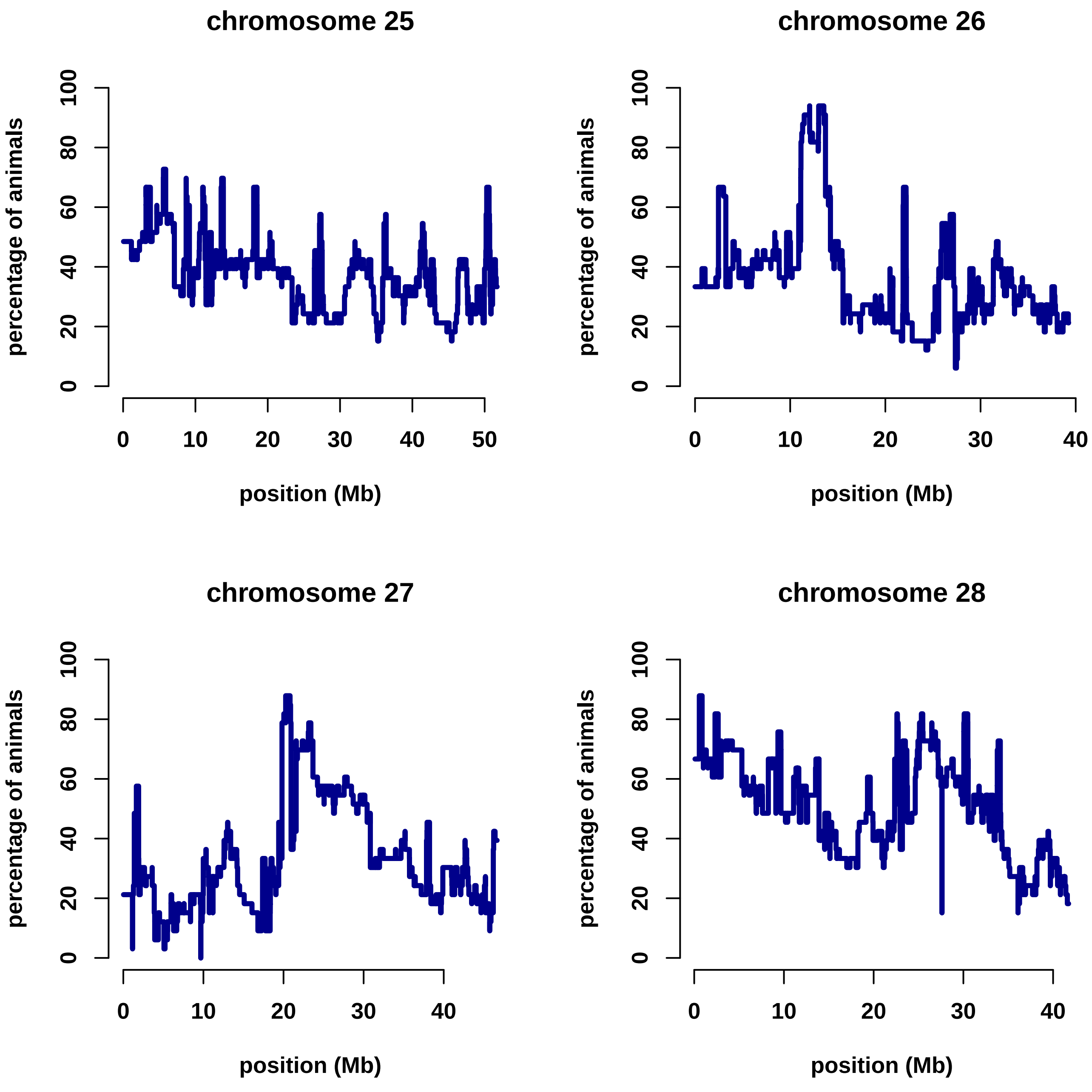
chromosome 25
percentage of animals
0
20
40
60
80
100
0
10
20
30
40
50
position (Mb)
chromosome 26
percentage of animals
0
20
40
60
80
100
0
10
20
30
40
position (Mb)
chromosome 27
percentage of animals
0
20
40
60
80
100
0
10
20
30
40
position (Mb)
chromosome 28
percentage of animals
0
20
40
60
80
100
0
10
20
30
40
position (Mb)

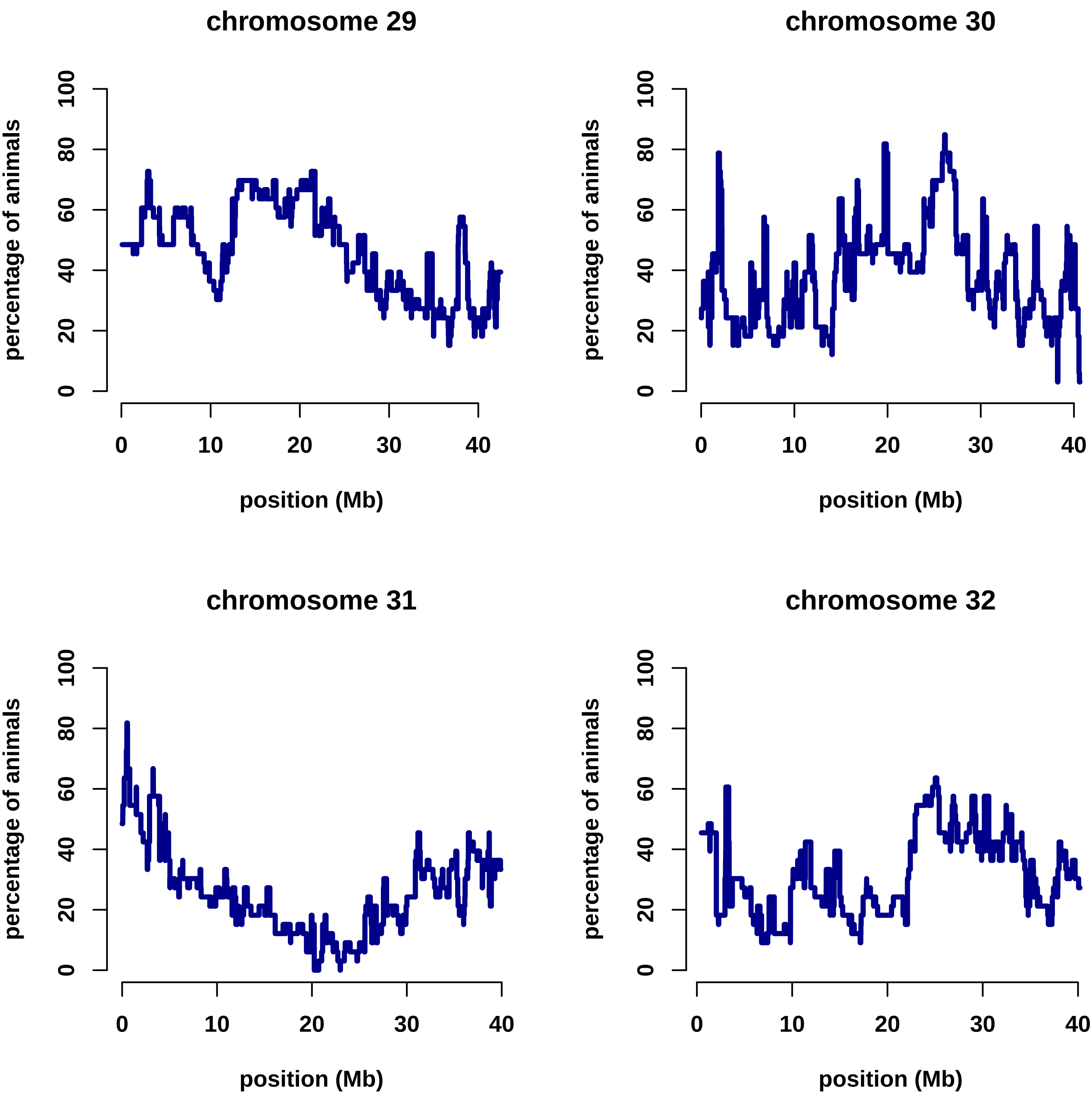
chromosome 29
percentage of animals
0
20
40
60
80
100
0
10
20
30
40
position (Mb)
chromosome 30
percentage of animals
0
20
40
60
80
100
0
10
20
30
40
position (Mb)
chromosome 31
percentage of animals
0
20
40
60
80
100
0
10
20
30
40
position (Mb)
chromosome 32
percentage of animals
0
20
40
60
80
100
0
10
20
30
40
position (Mb)

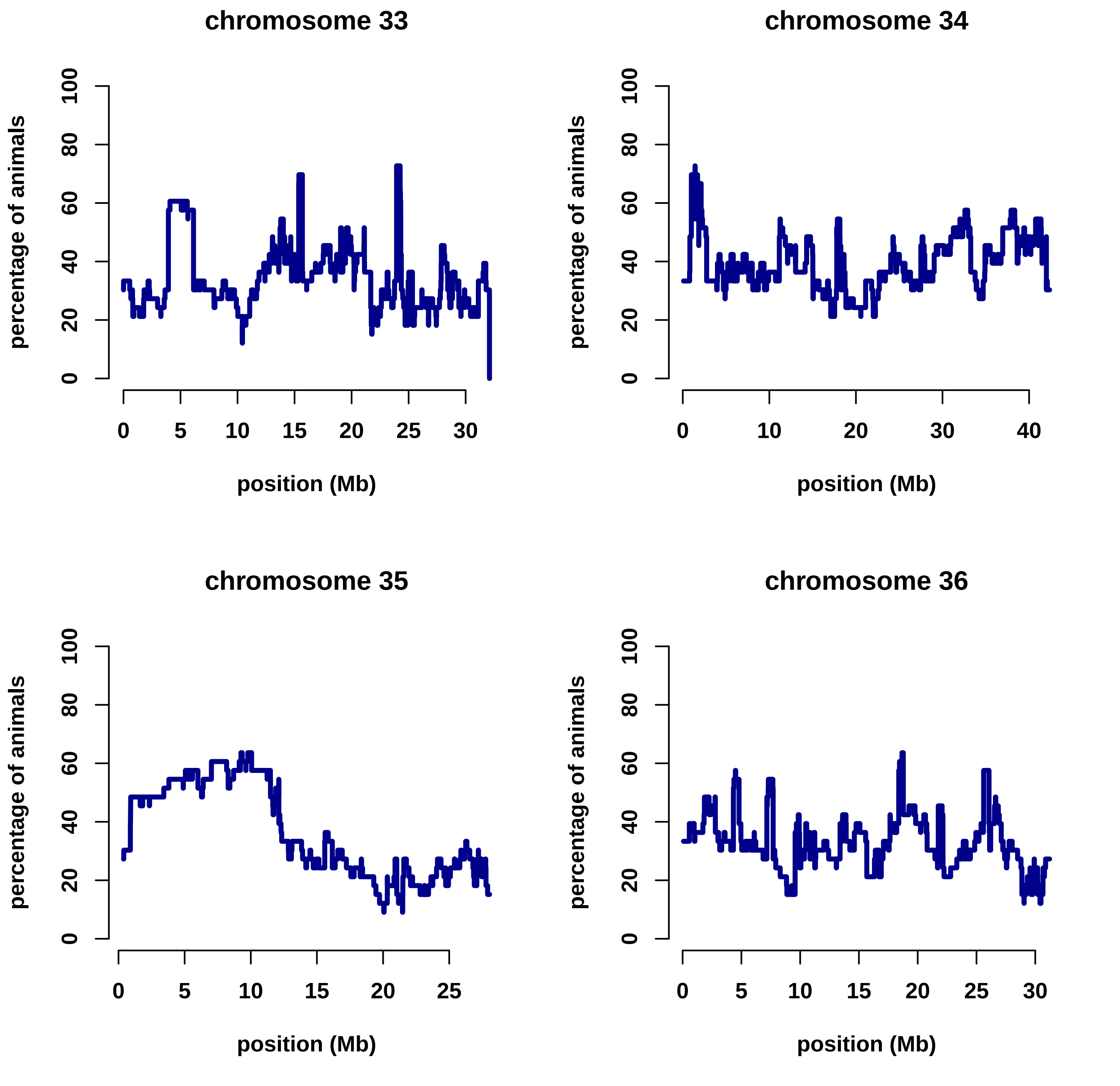
chromosome 33
chromosome 34
chromosome 35
chromosome 36
percentage of animals
position (Mb)
0
20
40
60
80
100
0
5
10
15
20
25
30
40

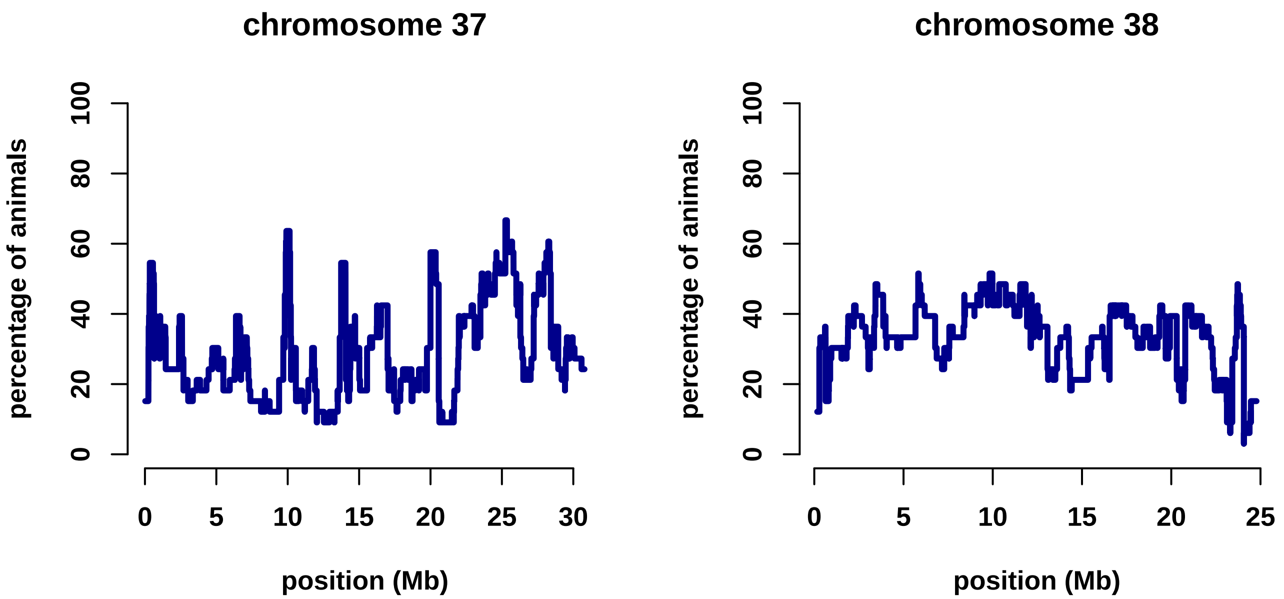
chromosome 37
chromosome 38
percentage of animals
position (Mb)
0
20
40
60
80
100
0
5
10
15
20
25
30
